# Legal and Regulatory Issues Governing Cannabis and Cannabis-Derived Products in the United States

**DOI:** 10.3389/fpls.2019.00697

**Published:** 2019-06-14

**Authors:** Alice Mead

**Affiliations:** GW Pharmaceuticals PLC, Cambridge, United Kingdom

**Keywords:** cannabidiol, cannabis, schedule I, rescheduling, controlled substances, PREEMPT, farm bill, HEMP

## Abstract

This chapter provides an in-depth discussion of the legal and regulatory frameworks surrounding cannabis in the United States, including federal law—as dictated by the Controlled Substances Act (CSA) and governed by various federal agencies like the FDA and DEA—as well as state law—as regulated by each state’s laws and regulations authorizing medical and/or adult use cannabis. First, the chapter discusses the definition and classification of cannabis under the CSA, including scheduling under the CSA as well as the process for and potentiality of removing cannabis from Schedule I. Then, it describes the activities relating to industrial hemp that are permitted under the 2014 and 2018 Farm Bill. Next, the chapter addresses state-level cannabis laws. The chapter also analyzes the question of whether state cannabis laws are invalidated and superseded by federal law. Moreover, this section examines the factors underlying the extent of the Department of Justice’s enforcement actions relating to state-authorized cannabis activities. The chapter then turns to CBD (cannabidiol) in particular, discussing CBD’s legal status under the CSA; the FDA’s role in regulating and approving CBD products for medical purposes; and the steps required to take an investigational CBD product through that approval process. The chapter concludes by contending that, while cannabis has had a long and twisting history, and although cannabis-derived products face daunting obstacles to achieving FDA approval as well as rescheduling under both federal and state law, the recent success of one product (Epidiolex^®^) should inspire other manufacturers to develop additional cannabis-derived products through the FDA process.

## A Brief History

Over the centuries, cannabis has been used for religious, industrial, therapeutic, and other purposes (Crowther et al., 2010; Potter, 2014). However, in the past 150 years, prominent social and political controversies involving cannabis have emerged around the world. Cannabis extracts and tinctures were widely prescribed in Europe and North America by physicians for a variety of medical conditions from the mid-1800s through the first few decades of the 20th century (Russo, 2004). However, in the United States in the early 1900s, smoked cannabis (then known by the slang term marijuana or marihuana) became associated with certain maligned ethnic and racial minorities, and many states prohibited its use (Bonnie and Whitebread, 1999; Schlosser, 1994). This ultimately resulted in the enactment of the federal Marihuana Tax Act of 1937 (Musto, 1972), which imposed taxes and other administrative burdens on both the medical and non-medical uses of cannabis.

In the United States in the years following the Act, and as the physician’s armamentarium expanded with new medication options, interest in the therapeutic effects of cannabis and cannabinoids waned until cannabis use increased in the 1960s, coincident, and indeed entwined, with antiwar and other social protest movements (Crowther et al., 2010). Young people around the United States experimented with cannabis and other drugs, and a number of them discovered that cannabis was helpful for certain medical conditions (Joy et al., 1999). In addition, research in Israel by Dr. Raphael Mechoulam demonstrated that tetrahydrocannabinol (THC) was the primary psychoactive component of the cannabis plant (Mechoulam et al., 1970).

These developments had several consequences. On the one hand, societal alarm over this increased use of cannabis reignited concerns about its deleterious effects and prompted research into its psychoactive and potentially addictive properties (The Medicalization of Cannabis, 2009). On the other, the concept of “medical marijuana” was born, and renewed interest in the medical properties of cannabis began slowly to emerge (Randall and O’Leary, 1998).

However, persistent negative attitudes about cannabis in certain countries, including the United States, culminated in the promulgation of the Single Convention on Narcotic Drugs (1961) (Mead, 2014). Under the Single Convention, cannabis and cannabis resin were placed in the most restrictive category^1^, and signatory parties were effectively required (subject to some flexibility for a party’s “good faith” determinations) to prohibit their manufacture, distribution, sale, etc. The United States was a party to the Single Convention, and, after the Marihuana Tax Act was struck down by the United States Supreme court in *Leary v.* United States [395 U.S. 6 (1969)], Congress enacted the Controlled Substances Act of 1970 (CSA), which consolidated all previous federal laws governing the handling of narcotics, stimulants, depressants, hallucinogens, etc. Title II of the Comprehensive Drug Abuse Prevention and Control Act of 1970, Pub. L. 91–513, 84 Stat. 1236.

## The Classification of Marijuana Under the Controlled Substances Act

The CSA was enacted in part to implement the United State’s obligations under the Single Convention. 21 USC 801(7). Its purposes were twofold: (1) it recognized that many controlled substances have a useful and legitimate medical purpose and are necessary to maintain the health and welfare of the public and (2) illegal importation, manufacture, distribution, and possession and improper use of such substances have a “substantial and detrimental effect” on public health and welfare. 21 USC 801 (1), (2). Under the CSA, substances are categorized into five schedules, depending on their therapeutic benefit and their potential to result in abuse, diversion, dependency, and addiction (Yeh, 2012). Schedule I is the most restrictive. Marijuana and tetrahydrocannabinols (THCs) are classified as hallucinogens in Schedule I, along with mescaline, peyote, psilocybin, MDMA, and LSD. 21 CFR 1308.11(d). Opium and virtually all opioids, coca leaves and cocaine, amphetamines, and a number of other substances are in Schedule II. 21 CFR 1308.12.

As a general rule, all substances, and the products containing or derived from such substances, are classified in the same schedule. However, there is a limited precedent for differential scheduling. For example, THC and its isomers are in Schedule I, but FDA-approved formulations of a THC isomer (delta-9) are in lower schedules. Compare 21 CFR section 1308.11(27) with 21 CFR section 1308.13(g)(1).

Under the CSA, marijuana is defined as:

The term “marihuana” means all parts of the plant *Cannabis sativa* L., whether growing or not; the seeds thereof; the resin extracted from any part of such plant; and every compound, manufacture, salt, derivative, mixture, or preparation of such plant, its seeds or resin. Such term does not include the mature stalks of such plant, fiber produced from such stalks, oil or cake made from the seeds of such plant, any other compound, manufacture, salt, derivative, mixture, or preparation of such mature stalks (except the resin extracted there from), fiber, oil, or cake, or the sterilized seed of such plant which is incapable of germination. 21 USC 802(16) (emphasis added).

As the definition indicates, marijuana includes its compounds and derivatives, as well as synthetic versions thereof. Therefore, the more than 100 (Brenneisen, 2007) cannabinoids found in the cannabis plant are also classified in Schedule I by operation of definition, and not as a result of a scientific analysis of their abuse potential. Only THC is separately and specifically listed in the CSA as a Schedule I substance.

Substances in Schedule I have no currently accepted medical use in the United States and a high potential for abuse. Schedule II substances similarly have a high potential for abuse, but they do have a currently accepted medical use. Schedules III–V substances have an accepted medical use and less (relative to each preceding schedule) abuse potential. 21 USC 812(b). Neither the CSA nor the Code of Federal Regulations (its implementing regulations) defines the concept of accepted medical use, but the United States Drug Enforcement Administration (DEA) has developed criteria that must be met in order to establish accepted medical use:

• The drug’s chemistry must be known and reproducible,
• There must be adequate safety studies,
• There must be adequate and well-controlled studies proving efficacy,
• The drug must be accepted by qualified experts, and
• The scientific evidence must be widely available (Drug Enforcement Administration, 1992).

The federal courts have thus far upheld DEA’s use of these criteria (Alliance for Cannabis Therapeutics vs. DEA, 1994). The existence of anecdotal reports of medical use (no matter how many) and the existence of state “medical marijuana” laws (no matter how many) are not sufficient to meet these criteria. However, FDA approval of a product as a prescription medication is sufficient (albeit not necessary) to demonstrate its accepted medical use. Grinspoon v. DEA, 828 F.2d 881(1st Cir. 1987).

Schedule I substances can be dispensed only in federally authorized research programs [Investigational New Drug (IND) authorized by FDA and DEA Schedule I research registration]. Schedule I status entails restrictive requirements for security, recordkeeping, storage, transport, and other activities. Schedule I substances cannot be imported into, or exported from, the United States, even for personal medical use, and even if the patient is enrolled in a clinical trial.

## Escaping From Schedule I

Rescheduling under federal law is generally conducted through an administrative process (Drug Enforcement Administration [DEA], 2010, 2016; Hoffman et al., 2018). Under this process, the FDA initially conducts a full assessment of the substance’s abuse potential, called an “eight-factor analysis” (8FA), because there are eight statutory factors that bear on abuse potential. 21 USC §811(c). DEA is bound by FDA’s medical and scientific determinations, but may consider additional data, such as the extent of abuse and diversion. DEA publishes a proposed rule in the Federal Register, which gives the public notice, and an opportunity to comment, object, or request an administrative law judge hearing. The DEA responds to the public’s comments and objections and then, if no persuasive request for a hearing has been made, publishes a Final Rule rescheduling the product or substance. If a hearing request is made and granted, this can delay the final rescheduling action for 2 years or more. 21 USC §811(j).

This rescheduling process can be initiated by DEA, by the Department of Health and Human Services/FDA as part of the new drug approval process, or by an interested person. 21 CFR 1308.44. Of course, Congress has the power to enact a law to schedule, reschedule, or entirely deschedule a substance. In doing so, Congress need not examine abuse of potential data or the results of an 8FA.

## The Status of Hemp Under Federal Law

Cannabis is an umbrella term, and numerous varieties—with different cannabinoid ratios or other content, such as terpene profiles—exist in nature or as a result of breeding. Informally, it could be said that cannabis varieties may be classified as either “drug-type” or as hemp. In Europe, there is a robust and well-established hemp industry (Vantreese, 2002; Commission of the European Communities, 2004). However, “hemp” is not actually defined under European law. Rather, certain pedigreed seed varieties may be cultivated, which have been bred historically for their fiber or seed, and which have a very low percentage of THC (not more than 0.2% by dry wieght) (Commission of the European Communities, 1989).

In the United States, the CSA does not define hemp. As indicated above, it defines marijuana, but certain parts of the marijuana/cannabis plant—stalk/fiber, sterilized seeds, and preparations thereof—are exempted from that definition. In other words, sterilized seeds and cannabis fiber (separated from the plant) are not marijuana and may be imported or otherwise used in commerce. However, there is an exception to the exemption: if “resin” is extracted from any part of the plant (including the excepted parts), that resin is still marijuana. Since all cannabinoids are located in resinous trichomes^2^ located on the inflorescences and upper leaves of the plant, in theory all extracts^3^ of cannabinoids from cannabis are defined as marijuana (Potter, 2014).

However, in December 2018, the 2018 “Farm Bill” [The Agriculture Improvement Act of 2018, Pub. L. 115-334 amending 21 USC §§802(16), 812(c)] was signed into law^4^. The 2018 Farm Bill defines hemp as the cannabis plant, or any part thereof, including its extracts and cannabinoids, having a THC concentration of not more than 0.3% on a dry weight basis. “Hemp,” as so defined, is removed from the definition of marijuana under the CSA and is no longer a controlled substance under federal law. The bill does not authorize interference with interstate commerce (although it does not affirmatively authorize such commerce); presumably, such commerce is lawful, at least between states that allow such commerce.

The 2018 Farm Bill requires hemp cultivation to be licensed and regulated pursuant to “state plans” promulgated by a state, which must contain, among other things, provisions for THC testing. If a state does not wish to issue a plan, the United States Department of Agriculture is authorized to do so. The USDA has authority to issue regulations and guidances, but the law explicitly preserves the existing jurisdiction of the FDA.

## The Development of State Cannabis Laws

In 1996, during the AIDS crisis in California, the voters approved an initiative to decriminalize certain cannabis-related activities by specific categories of persons. Proposition 215, the Compassionate Use Act of 1996, allowed a qualifying patient and his/her caregiver to cultivate and possess cannabis for medical purposes. CA Health and Safety Code, Article 2 (Cannabis), §11362.5. Oregon and Washington followed shortly thereafter. Medical use was limited in the years that immediately followed, since many patients and caregivers were not able to cultivate their own cannabis, and many physicians were unwilling to provide the “recommendations” necessary to qualify the patient for legal protection. However, beginning in about 2004, retail dispensaries began to appear, as well as larger numbers of physicians who were willing to provide recommendations. In 2012, Colorado became the first state to approve, by initiative, the recreational or “adult use” of cannabis. Amendment 64 (Use and Regulation of Marijuana); Article 18, §16 of the Colorado Constitution.

Fast forward to today. Thirty-three states and the District of Columbia have enacted laws allowing the use of cannabis for therapeutic purposes (NCSL). Eleven states and the District of Columbia permit recreational or “adult use” of cannabis (ProCon, 2018b). Seventeen additional states only permit products that are high in cannabidiol (CBD) and low in THC^5^.

The provisions of medical cannabis laws vary significantly by states (ProCon, 2018a). In most states with medical use laws, physicians, and sometimes other types of health care providers, must recommend that the patient use cannabis or advise that the patient might benefit from such use (because cannabis is a Schedule I substance, physicians cannot prescribe it). Physicians are often exempt from professional and other liability that is premised solely on the fact that they issued such a recommendation or advice. However, physicians can still be liable for issuing recommendations in a manner that falls outside the standard of care (Medical Board of California, 2018) or that aids and abets a violation of federal law (Conant v. Walters, 2002). The medical use laws generally include a list of “qualifying” medical conditions, with which a patient must be diagnosed. These lists may be derived from published scientific studies or case reports, from testimony of individuals or advocacy groups, or other sources. While they vary, these lists often contain conditions like epilepsy and cancer.

Similar to the medical laws, adult use laws vary by state as well (ProCon, 2018a,b). Apart from Vermont and D.C., which do not allow commercial sales—the other ten states allowing commercial activity established regulatory systems that allow for possession and personal cultivation as well as commercial cultivation and sales. All recreational states require individuals possessing or cultivating cannabis to be 21 or over. The quantities of cannabis an individual can possess range by state (generally around an ounce or two), and so do the number of plants one can have (generally up to six). Across the recreational states, medical marijuana laws are, overall, more permissive regarding individual possession and cultivation, as they often permit patients to purchase and cultivate larger quantities as well as access more potent products and enjoy a lower tax rate.

In terms of commercial systems, the 10 states that permit it feature differing regulatory systems, but generally allow for state-licensed businesses to engage in commercial production, distribution, and sales of cannabis and cannabis products. Additionally, different states have different methods of regulating their medical and recreational systems. California, for instance, features a singular, harmonized regulatory framework—the Medicinal and Adult-Use Cannabis Regulation and Safety Act (SB 94) (CA Business and Professions Code [BPC], 2016) [need the full statutory citation in the code]—but divides medical and recreational into separate market streams. Somewhat differently, Colorado has separate constitutional amendments for each system, while also dividing medical and adult use into separate market streams.

Last, the quality control (including testing) and label requirements for both medical and recreational are quite uneven and may be non-existent in some states (Klieger et al., 2017). Some states—like California (recreational and medical)—require laboratory testing of cannabis and cannabis products to make sure that they meet quality and safety standards, while other states—such as Arizona (just medical)—do not have state-mandated testing (Milley, 2018). Since, for prescription medications, these requirements are generally determined by FDA, it may be challenging for states to develop such requirements and to find adequate resources to enforce them. However, a number of international standard-setting organizations, such as ASTM and AOAC, are engaged in developing standards for the testing, quality control, etc., of cannabis and cannabis products. Several cannabis quality control guidance documents are available from the American Herbal Products Association and from American for Safe Access, and these are being employed by a number of states to establish quality standards.

## State and Federal Law Conflict?

Under the Supremacy Clause of the United States Constitution, federal law preempts or supersedes state laws that are inconsistent, or in conflict, with federal law in certain ways (Todd, 2012; Mead, 2014). However, there is a specific provision in the federal CSA that states that state drug laws are only preempted if there is an “affirmative conflict” with the CSA. Indeed, state law is the primary enforcement authority for drug-related offenses.

The state cannabis laws described above—particularly the early laws—can be said merely to decriminalize certain cannabis-related activities under state criminal laws. They do not require private individuals or businesses to conduct cannabis-related activities. If an individual/business wishes to avoid a violation of the federal CSA, that person or entity can simply avoid cannabis-related activities altogether. As a result, most state and federal courts that have considered this issue have found that these state laws are not invalidated by the CSA (Brilmayer, 2017; Guenthner, 2017).

Individuals and entities who choose to engage in cannabis-related activities would violate the federal CSA. However, the federal government generally does not prosecute individuals who possess (or share) small amounts of cannabis, instead focusing their enforcement priorities on larger cannabis commercial entities or drug trafficking organizations, particularly those involved in interstate transport or foreign importation (see section “Reasons for Limited Federal Enforcement of the Controlled Substances Act”).

## Reasons for Limited Federal Enforcement of the Controlled Substances Act

Under the Obama administration, the Department of Justice (DOJ) took a less aggressive stance toward cannabis-related activities than it had under previous administrations. In 2013, DOJ issued a memorandum intended to guide United States attorneys in the exercise of enforcement discretion (Cole, 2013). The memo essentially stated that it was not a DOJ priority to take enforcement action against persons or entities involved in cannabis activities if those activities were lawful under state cannabis laws (whether medical or recreational). However, DOJ would consider enforcement action if those activities negatively impacted eight specific federal interests^6^. This memo also applied to the cultivation and manufacture of hemp outside of the authority of the Farm Bill (US Attorney Marshall Letter to Rep. Blumenauer, 2018).

Former Attorney General Jeff Sessions rescinded this memo (Sessions, 2018). However, no notable enforcement action has been taken. This may be a result of other factors. An amendment to the Consolidated Appropriations Act of 2018 prohibits the DOJ from using any funds to prevent states from implementing their medical (not adult use) marijuana laws and prevents DOJ/DEA and other federal agencies from using funds to prevent hemp-related activities that are lawful under the Farm Bill. 115th Congress, Pub. L. No. 115-141. This Appropriations Act is valid through September 2018 but is likely to be extended by one or more Continuing Resolutions. In addition, there are Members of Congress who, for various reasons, would likely oppose significant DOJ/DEA enforcement against state-authorized cannabis activities. Finally, the country is facing a prescription drug abuse crisis—largely involving opioids—and DOJ/DEA have other enforcement priorities. These factors may explain the lack of aggressive enforcement of the CSA against cannabis-related activities. The current Attorney General William Barr has indicated that he will follow the spirit of the Cole memo (Angell, 2019).

## The Emergence of Cannabidiol

Public interest in cannabidiol (CBD) has exploded in the past few years. CBD can be purchased online, in cannabis dispensaries, and, increasingly, in grocery and natural foods stores, and other retail outlets. How did CBD emerge into the public eye?

Unlike THC, CBD does not have euphoriant properties (Pertwee, 2004). Although the identity and structure of CBD have been known for decades, limited research had been conducted to explore its therapeutic potential. Preclinical studies suggested a wide range of potential applications (Pertwee, 2004), but clinical studies in several indications, including epilepsy, had produced uneven and unconvincing results. In 2003, researchers at the National Institutes of Health (NIH) secured a patent claiming a method of treating diseases caused by oxidative stress, such as neurodegenerative or ischemic disease, by the administration of non-psychoactive cannabinoids (Hampson et al., 2003).

In 2007, the laboratory of Professor Ben Whalley conducted a series of preclinical studies that robustly demonstrated that CBD had anti-seizure properties (Jones et al., 2010, 2012). Once disseminated at scientific conferences and published, these studies caused a great deal of interest in the United States. A small non-profit, Project CBD, was formed, which publicized the results (Project CBD, 2018). Cannabis growers, who had inadvertently discarded CBD-rich varieties in the effort to breed varieties rich in THC, took note. A newly established analytical testing laboratory examined plant samples and determined that some CBD-rich varieties still remained, and a few extracts were made. The Discovery Channel in 2011 filmed one parent administering a CBD extract to his son who had a catastrophic form of epilepsy (Discovery Channel, “Weed war chronicles”), and word traveled in the community of parents with children with similarly intractable epilepsies.

A California family, learning about CBD from their nurse, tried several types or products with their son who had an intractable epilepsy. Unfortunately, he had had a very uneven response to those products. Upon reading the recent preclinical research, they realized that GW Pharmaceuticals, the sponsor of the research, had a standardized form of CBD, and they undertook to contact the company to request access to the product (Vogelstein, 2015).

A Colorado family, who had seen the Discovery Channel segment on YouTube, also searched for CBD for their daughter who also had a devastating type of epilepsy. They located a local source of CBD, which significantly reduced their daughter’s seizures (Maa and Figi, 2014). Her dramatic response was captured in August 2013 in a documentary entitled “Weed,” produced by Dr. Sanjay Gupta of CNN. The program unleashed a tidal wave of interest among families with similarly afflicted children. Families moved to Colorado in search of access to the product that came to be known as “Charlotte’s Web”; states passed laws permitting possession and sometimes manufacture of high-CBD, low-THC products, and within a few years, a wide variety of CBD products were available, purporting to treat a multitude of medical conditions.

## Sources of Cannabidiol

As indicated above, over 100 cannabinoids are found in the plant. The cannabis plant (including hemp varieties) produces cannabinoids in glandular trichomes, which resemble little golf balls, often on a small stalk. These trichomes are concentrated in the inflorescences and, to a more limited extent, in the upper leaves (Potter, 2013, 2014). The stalk and seeds have essentially no cannabinoids (Wassem et al., 2018)^7^. Hence, although hemp seed oil offers a good source of Omega 3 and 6 fatty acids, it contains effectively no cannabinoids.

THC and CBD are the most prevalent cannabinoids. Beginning in the 1970s, cannabis growers began to breed cannabis varieties that expressed ever-increasing concentrations of THC, since most people believed that all of the effects of cannabis—both psychoactive and therapeutic—lay in the THC. When CBD was “rediscovered” in the United States, as described above, the “CBD-rich” varieties that were available to be extracted were “drug-type” varieties, rather than classic hemp varieties. Subsequently, in the wake of the 2014 Farm Bill, hemp varieties became the primary source of CBD.

Classic hemp varieties, i.e., those originating in Europe, are not efficient sources of CBD. The original varieties contained 0.5–4.0% CBD by dry weight (European Hemp Industries Association, 2018), although, as a result of breeding, newer varieties may contain as much as 7–8% CBD (Lee, 2016). Even at that higher level, a large quantity of hemp must be cultivated in order to extract a meaningful amount of CBD. Since hemp is a “phytoremediator,” i.e., it absorbs heavy metals from the soil (Cascardi, 2018), it is essential that the conditions of cultivation be carefully controlled.

Cannabidiol may still be derived from drug-type varieties of cannabis and then purified to remove some or all of the THC. Alternatively, CBD may be manufactured via a synthetic process. However, in that case, it is important that the manufacturer select an appropriate synthetic process that produces the same CBD isomer as that produced by the plant. A different isomer could have a very different therapeutic and/or toxicological profile (Hanus et al., 2005).

## Legal Status of Cannabidiol Under the Controlled Substances Act

As indicated above, CBD is classified in Schedule I of the CSA because it is considered a compound or derivative of cannabis/marijuana. 21 USC 802. However, as indicated above, the 2018 Farm Bill has descheduled hemp as it is defined under that law. Therefore, commercial activity with hemp (including its extracts and cannabinoids) is now lawful. A DEA registration is no longer required to cultivate hemp or to conduct research with hemp. However, if clinical research, i.e., involving human subjects, is involved, an investigational new drug exemption (IND) must still be opened with FDA, and the investigational product must be manufactured in a facility that complies with good manufacturing practice (GMP) requirements.

## Cannabidiol and the FDA

The Food, Drug and Cosmetic Act (FDCA) prohibits any product from being sold in interstate commerce if it is intended to be used in the treatment, mitigation, diagnosis, or cure of a disease or a disorder—unless that product has been approved by FDA as a prescription medication. 21 USC section 321(g)(1). In determining “intended use,” FDA will examine a wide variety of sources—labels, advertisements, websites, social media—to ascertain a product’s intended use (FDA, 2018b). In 2015–2018, FDA has sent warning letters to manufacturers of CBD products (sold online and in other retail outlets), informing them that their products were misbranded and hence illegal as a result of medical claims (FDA, Warning Letters and Test Results for Cannabidiol-Related Products; FDA, 2018b).

In addition, in 2015 and 2016, FDA tested many of the CBD products and determined that more than 90% of them contained much less CBD than the labeled amount, some had no CBD at all, and some had greater amounts of THC (US FDA, Warning Letters and Test Results for Cannabidiol-Related Products). This quality-control concern has been affirmed by a study of CBD products sold in dispensaries (Bonn-Miller et al., 2017).

In 2018, FDA issued the first CBD Warning Letter that relied in part on deficiencies in Good Manufacturing Practices (for pharmaceutical products, not for dietary supplements) (FDA, 2018b). FDA also targeted, for the first time, topical products for which medical claims were being made.

Furthermore, beginning in 2016, FDA stated in its Warning Letters that CBD cannot be sold as an ingredient in a food or dietary supplement. FDA relied on sections 21 USC 201(ff)(3)(B)(ii) and 21 USC 321(ff)(3)(B)(ii) of the FDCA, which provide that, if a substance is being studied in substantial clinical trials [i.e., as part of a new drug application (NDA) process], a different manufacturer cannot attempt to do a “shortcut” around the lengthy and expensive NDA process by incorporating the substance into a food or dietary supplement. The only exception to this prohibition is for a substance that was already being marketed as a food or dietary supplement before the clinical trials began. The substance must have been overtly marketed, that is, not merely present as an unlabeled impurity. An argument can also be made that the marketing must not have been violative of a federal law like the CSA.

FDA considered the evidence and determined that CBD had been studied initially under an investigational new drug exemption (IND) in 2006 and again in 2014, and that CBD had not been marketed as a food or dietary supplement before that time (FDA, 2018a). Immediately after the 2018 Farm Bill was signed into law, Then-FDA Commissioner Gottlieb issued a statement emphasizing that, while hemp and cannabinoids derived from it are no longer scheduled substances, CBD and THC cannot lawfully be sold in food or in dietary supplements. The Commissioner did note that, under the above provisions, FDA has authority to issue a regulation allowing a substance to be marketed in food or dietary supplements and that the agency would hold a public meeting to take input from stakeholders on whether it should pursue such a process (Gottlieb, 2018, That meeting took place on May 31, 2019).

A number of manufacturers are apparently attempting to avoid FDA’s statement concerning section 321(ff)(3)(B)(ii) by marketing their products as “hemp extracts” (Mister, 2019). However, many of these products still provide the CBD content on the label, website, or certificate of analysis (COA). It remains to be seen whether FDA will determine that these products are violative of the FDCA.

## How Can a Cannabis-Derived Product Go Through the FDA Approval Process?

Media reports on cannabis often include the contention that, since it is a Schedule I substance, cannabis (and its derivatives) cannot be researched in the United States, much less move successfully through the rigors of the FDA approval process. This statement is, for the most part, false.

Schedule I status certainly increases the level of complexity for any research study. For example, all researchers—whether preclinical or clinical—must obtain Schedule I research registrations. 21 CFR section 1301.18. By contrast, researchers who have DEA Practitioner registrations in Schedules II–V (which most physicians would have) may conduct research in Schedules II–V as a “coincident activity” to their Practitioner registrations and do not need to secure any additional registrations or licenses. 21 CFR section 1301.13. Since cannabis is a controlled substance, a researcher cannot obtain cannabis from dispensaries or from patients in order to test the therapeutic effects of varieties that patients may be using. The cannabis must come from a cultivator who is registered with DEA as a Schedule I manufacturer. In other words, a researcher with a Schedule I research registration must obtain cannabis from another DEA registrant.

In addition to the DEA Schedule I registration, researchers must generally also obtain Schedule I research licenses from the state-controlled drugs authority. The application process for these Schedule I registrations/licenses, including research site inspections, generally do not take place concurrently, but rather are sequential, with the state usually going first.

Furthermore, the University of Mississippi is currently the only federally lawful United States source of research-grade cannabis. The United States “single source” position has historically been based on its perceived obligations under the Single Convention on Narcotic Drugs, 1961. Under the Single Convention, if a signatory country affirmatively authorizes the domestic cultivation of cannabis, the cannabis stocks must be exclusively owned and controlled by a national agency. The United States national agency is the National Institute on Drug Abuse (NIDA) part of NIH. NIDA contracts with the University of Mississippi to produce research-grade cannabis. Even academic researchers who are conducting investigator-initiated trials (IITs) must secure research cannabis through NIDA.

This single-source requirement is a particular problem for manufacturers since those who wish to conduct United States research on a cannabis-derived product that will lead to an NDA (including Phase 1–3 research and the necessary body of preclinical safety and toxicology studies) must be able to cultivate a large quantity of a specific variety of cannabis under the same consistently controlled conditions. The investigational material used in the Phase 3 studies must be the same as that used in the toxicology studies, or bridging studies must be conducted. The Phase 3 material must be the same as that used in the commercialized product (FDA, 2016). The typical annual outdoor yield from the University of Mississippi 12 acre “farm” is 500 kg of plant material (University of Mississippi, 2018. Marijuana Research). By way of comparison, in order to produce enough material for Phase 3 clinical trials and commercialization of its CBD product Epidiolex^®^, GW Pharmaceuticals cultivates a high-CBD expressing chemovar in a 45-acre glasshouse.

Drug enforcement administration announced in 2016 that it would register additional cultivators to produce research-grade cannabis, as well as cannabis to be used in the manufacture of FDA-approved, cannabis-derived products, but thus far, no registrations have been issued (Drug Enforcement Administration [DEA], 2016).

However, this national agency requirement applies only to cannabis that is cultivated within that country’s border. Investigational cannabis products may be manufactured outside the country and, in the United States, imported under an IND for purposes of research. Two cannabis-derived products (Sativex^®^and Epidiolex^®^)^8^ were researched in the United States in this manner, and United States researchers have recently been permitted by DEA to import cannabis capsules from Canada for purposes of research (Johnston).

Of course, any cannabis-derived investigational product must demonstrate quality, safety, and efficacy in order to achieve FDA approval. Putting aside the hurdles described above, a complex cannabis product, i.e., comprised of major and minor cannabinoids, as well as terpenes and flavonoids, faces significant standardization and quality control issues. It is important to build quality into the botanical starting materials. Outdoor cultivation can introduce the risk of contamination from adjacent pesticide and synthetic fertilizer use, bird droppings, etc. In order to ensure consistency in cannabis content, plants should be propagated by clones or some similar process, rather than seeds. The growth medium should be devoid of heavy metals. Ideally, no pesticides or fungicides would be used. Specifications for the botanical raw material (BRM), botanical drug substance (BDS) (the processed or extracted material), and the finished botanical drug product (BDP) must be set and agreed upon by the FDA. Since cannabinoids are present almost exclusively in the acid form (THCA and CBDA) in the plant, the material must undergo decarboxylation to remove a carboxyl group, if the neutral form (THC and CBD) is desired. This decarboxylation step can be challenging to conduct properly—without leaving incompletely decarboxylated material or degrading the cannabinoids—particularly on a large commercial scale (Wang et al., 2016). If the dosage form requires extraction of the cannabinoids, it is important that the extraction process does not result in a BDS with residual dangerous solvents. If the finished product will be composed of a single cannabinoid, a complex crystallization process is required (Wang et al., 2016). Stability studies on both the BDS and BDP must support the expiration date, usually 2–3 years (Ng, 2015).

FDA has issued a guidance to assist sponsors in developing botanically complex prescription medications (US Dep’t of Health and Human Services and US FDA, 2016). While this guidance allows some flexibility in the early stages of research, by the time the product reaches Phase 3, the requirements are essentially the same as for any product composed of a single synthetic molecule. If the product is composed solely of a purified cannabinoid, it is subject to all such requirements.

As with any investigational product, the FDA will inspect all manufacturing sites and processes to ensure that a Quality Management System is in place and that all current good manufacturing practices (cGMP) for pharmaceutical products are being followed (Ng, 2015). This inspection is very extensive and can take 5–7 business days.

Both a BDP and a purified cannabinoid product must undergo a full range of preclinical and clinical safety and efficacy testing, including drug/drug and food/drug interaction studies. In addition, because a cannabinoid product is derived from the cannabis plant and is therefore generally considered to be active in the central nervous system, the product must go through a battery of tests to determine the extent (or not) of its abuse potential: receptor binding and preclinical studies, as well as a special human abuse liability study.

As part of the NDA, the manufacturer/sponsor will analyze these studies and make a rescheduling proposal to FDA. FDA will assess these data and, shortly before or after the product is approved, FDA will make a rescheduling recommendation to DEA. Under the recent Improving Regulatory Transparency in New Medical Therapies Act, 21 USC section 811(j), DEA has 90 days within which to evaluate all data and make a rescheduling decision, which is published in the Federal Register in the form of an interim final rule (IFR). Under the IFR, the product may be sold.

Drug enforcement administration will subsequently conduct the full administrative rescheduling process described earlier, with public notice and opportunity to comment, object, or request an administrative law judge hearing. It is unlikely (but possible), at the completion of this process, that DEA would modify the schedule, since all material scientific evidence would presumably already have been considered by the agencies in the initial rescheduling action. However, if an international treaty requires a specific scheduling placement, DEA will issue a Final Rule (not an IFR or a Proposed Rule) rescheduling the product^9^.

If this were any other NCE product (usually comprised of a single synthetic molecule), the IFR would effectively mark the end of the process, and the product would be available to be marketed in all the states. Having been scheduled for the first time by the DEA during the NDA process, the NCE product is not yet scheduled under state law. Since it is unscheduled, it may be prescribed by physicians and dispensed by pharmacies.

However, this is not true for cannabis-derived products. Virtually all of the states have adopted their own version of the federal CSA (Uniform Controlled Substances Act, 1994), and marijuana and its derivatives are in Schedule I under most of those state laws (even in states with adult use and/or medical access laws). Few states automatically change the schedule of a product or substance merely because the DEA has done so. The rest either require that rescheduling be conducted by a state agency through a sometimes-prolonged administrative process or by legislation enacted by the state legislature, and many legislative sessions occur only during the first 4 months of the year or every other year (National Council of State Legislatures [NCSL], 2018). This can delay patient access to a new cannabis-derived product by as much as 2 years in many states (American Medical Association, 2018).

## Conclusion

Cannabis has traveled a long and twisting road across the centuries. Its social acceptability is gradually increasing around the world. In the United States, significant legal changes have occurred; at the state level, cannabis is legal for some medical purposes in 47 states and legal for adult use in 11 of those. However, cannabis and its cannabinoids are classified in Schedule I of the federal CSA, which imposes strict controls on possession, manufacturing, distribution, and dispensing. Schedule I substances may be dispensed only in a federally authorized research program, and cannabis used for research must be obtained only from the University of Mississippi. The 2018 Farm Bill has removed hemp and its extracts (as defined) from the schedules of the CSA, thereby facilitating research and commercial activity with hemp. Nevertheless, the FDA has indicated that CBD and THC cannot be lawfully sold as an ingredient in foods or dietary supplements under the FDCA, although the FDA is currently considering the possibility of creating a lawful regulatory pathway for such products. Developing cannabis-derived products into prescription medications faces some unique research challenges. However, on June 25, 2018, FDA approved Epidiolex^®^, a highly purified, plant-derived CBD product, for the treatment of seizures associated with two types of devastating childhood-onset epilepsies, Dravet syndrome and Lennox–Gastaut syndrome, in patients 2 years and older. Hopefully, the success of Epidiolex^®^will encourage other manufacturers to bring additional cannabis-derived products through the FDA process, thereby increasing treatment options for patients.

## Author Contributions

The author confirms being the sole contributor of this work and has approved it for publication.

## Conflict of Interest Statement

AM is an employee of Greenwich Biosciences, which manufactures Epidiolex, an FDA approved prescription cannabidiol (CBD) medication.

## Footnotes

1 The Expert Committee on Drug Dependence of the World Health Organization has recently recommended, among other things, that cannabis and cannabis resin be removed from the most restrictive schedule of the Single Convention (Schedule IV) and be retained in Schedule I, a less restrictive schedule, suggesting that WHO/ECDD has concluded that these materials have medical potential as well as abuse potential. These recommendations may be considered by the Commission on Narcotic Drugs at its March 2019 meeting. WHO, 41st Report of the Expert Committee on Drug Dependence Annex 1n (2018). https://mjbizdaily.com/wp-content/uploads/2019/01/Annex-1-41-ECDD-recommendations-cannabis-22Jan19.pdf.

2 A trichome is the small epidermal appendage that exists on the plant vegetation where cannabinoids, terpenes, and flavonoids are produced.

3 The DEA has created a separate Schedule I category for “marihuana extracts.” All synthetic copies of botanical cannabinoids, and cannabis itself, remain in the category “marihuana.”

4 Under the previous 2014 Farm Bill [section 7606 of the Agriculture Act of 2014, (Public Law 113–79), 7 U.S. C. 5940 (2014)], state departments of agriculture and institutions of higher learning were authorized to conduct research with industrial hemp.

5 See National Conference on State Legislatures [NCSL] (2018), supra.

6 According to the Cole memo, these eight enforcement priorities include preventing the distribution of marijuana to minors; preventing revenue from the sale of marijuana from going to criminal enterprises, gangs, and cartels; preventing the diversion of marijuana from states where it is legal under state law in some form to other states; preventing state-authorized marijuana activity from being used as a cover or protect for the trafficking of other illegal drugs or other illegal activity; preventing violence and the use of firearms in the cultivation and distribution of marijuana; preventing drugged driving and the exacerbation of other adverse public health consequences associated with marijuana use; preventing the growing of marijuana on public lands and the attendant public safety and environmental dangers posed by marijuana production on public lands; and preventing marijuana possession on or use on federal property.

7 Cannabinoids were recently detected in the root.

8 Sativex^®^is approved in over 25 countries outside the United States, and Epidiolex^®^was approved by the FDA in June 2018 for the treatment of seizures associated with two types of rare, serious, childhood-onset epilepsies.

9 This is what occurred with Epidiolex^®^, a pharmaceutical formulation of plant-derived, highly purified cannabidiol (CBD). The DEA issued a Final Rule placing botanically derived CBD, when containing not more than 0.1% THC, and when incorporated into an FDA-approved product, into Schedule V. 83 Fed. Reg. 48950 (September 28, 2018).

## References

[B1] Alliance for Cannabis Therapeutics vs. DEA (1994). *15 F.3d 1131 (D.C. Cir.1994).*

[B2] American Medical Association (2018). *American Medical Association, Resolution 502A.* Available at: https://www.ama-assn.org/sites/default/files/media-browser/public/hod/a18-reference-committee-reports.pdf (accessed October 16 2018).

[B3] Angell T. (2019). *Trump Attorney General Pick Puts Marijuana Enforcement Pledge in Writing.* Available at: https://www.forbes.com/sites/tomangell/2019/01/28/trump-attorney-general-pick-puts-marijuana-enforcement-pledge-in-writing/ (accessed January 28 2019).

[B4] Bonnie R. Whitebread C. (1999). *The Marijuana Conviction: A History of Marijuana Prohibition in the United States.* Springfield: Bookworld Services.

[B5] Bonn-Miller M. O. Mallory J. E. L. Brian F. T. Jahan P. M. Travis H. Vandrey R. (2017). Labeling accuracy of cannabidiol extracts sold online. *JAMA* 318 1708–1709. 29114823 10.1001/jama.2017.11909 PMC5818782

[B6] Brenneisen R. (2007). “Chemistry and analysis of phytocannabinoids and other *Cannabis* constituents,” in *Marijuana and the Cannabinoids*, ed. Elsohly M. (New York, NY: Humana Press).

[B7] Brilmayer L. (2017). *A General Theory of Preemption: With Comments on state Decriminalization of Marijuana.* Available at: https://lawdigitalcommons.bc.edu/bclr/vol58/iss3/4/. (acessed June 12 2017).

[B8] CA Business and Professions Code [BPC] (2016). *Business, and Professions Code, Division 10 (Cannabis, 26000–26231.2).* Napa County, CA: BPC.

[B9] Cascardi L. (2018). *Hemp (Cannabis sativa L),” in as a Phytoremediator.* Available at: https://www.votehemp.com/wp-conte: nt/uploads/2018/09/Phytoremediation-with-hemp-by-Laura-Cascardi.pdf (accessed September 23 2018).

[B10] Cole J. (2013). Guidance regarding marijuana enforcement. *Fed. Sent. Report.* 26 217–220. 10.1525/fsr.2014.26.4.217

[B11] Commission of the European Communities (1989). *Commission Regulation (EC) No 1782/2003 Establishing Common Rules for Direct Support Schemes Under the Common Agricultural Policy and Establishing Certain Support Schemes for Farmers.* Brussels: European Union.

[B12] Commission of the European Communities (2004). *Commission Regulation (EC) No 796/2004 Laying Down Detailed Rules for the Implementation of Cross-Compliance, Modulation, and the Integrated Administration and Control System Provided for in Council Regulation (ED) No 1782/2003 Establishing Common Rules for Direct Support Schemes Under the Common Agricultural Policy and Establishing Certain Support Schemes for Farmers.* Brussels: European Union.

[B13] Conant v. Walters (2002). *309 F.3d* 629 (9th Cir. 2002).

[B14] Crowther S. M. Reynolds L. A. Tansey E. M. (eds) (2010). *The Medicalization of Cannabis Wellcome Witnesses to Twentieth Century Medicine*, Vol. 40 London: Wellcome Trust Centre for the History of Medicine, 4–5.

[B15] Drug Enforcement Administration [DEA] (2010). *Listing of Approved Drug Products Containing Dronabinol in Schedule III.* Available at: https://www.federalregister.gov/documents/2010/11/01/2010-27502/listing-of-approved-drug-products-containing-dronabinol-in-schedule-iii (accessed September 24 2018).

[B16] Drug Enforcement Administration [DEA] (2016). Applications to become registered under the controlled substances act to manufacture marijuana to supply researchers in the United States. *Fed. Regist.* 81 53846–53848. 27529905

[B17] Drug Enforcement Administration (1992). *Final Order of the Administrator of the Drug Enforcement Administration.* Available at: https://www.gpo.gov/fdsys/pkg/FR-1992-03-26/pdf/FR-1992-03-26.pdf (accessed September 24 2018).

[B18] European Hemp Industries Association (2018). *Position Paper of the European Industrial Hemp Association (EIHA) on: Reasonable Regulation of Cannabidiol (CBD) in Food, Cosmetics, as Herbal Natural Medicine and as Medicinal Product.* Available at: http://eiha.org/media/2016/10/18-10-EIHA-CBD-position-paper.pdf (accessed October 2018).

[B19] FDA (2016). *Botanical Drug Development Guidance for Industry.* Available at https://www.fda.gov/downloads/Drugs/Guidances/UCM458484.pdf (accessed September 23 2018).

[B20] FDA (2018a). *Inspections, Compliance, Enforcement, and Criminal Investigations.* Available at: https://www.fda.gov/ICECI/EnforcementActions/WarningLetters/ucm616278.htm (accessed September 24 2018).

[B21] FDA (2018b). *Warning Letters and Test Results for Cannabidiol-Related Products.* Available at: https://www.fda.gov/ICECI/EnforcementActions/WarningLetters/2017/ucm583192.htm (accessed September 23 2018).

[B22] Flockhart I. Wheatley G. Dring S. Archer L. (2019). *Methods of Purifying Cannabinoids From Plant Materials.* Available at: https://patents.google.com/patent/US20050266108 (accessed March 19 2019).

[B23] Gottlieb S. (2018). *Statement from FDA Commissioner Scott Gottlieb, M.D., on signing of the Agriculture Improvement Act and the Agency’s Regulation of Products Containing Cannabis and Cannabis-Derived Compounds.* Available at: https://www.fda.gov/NewsEvents/Newsroom/PressAnnouncements/ucm628988.htm?utm (accessed December 20 2018).

[B24] Guenthner F. R. (2017). *Pot, Printz, and Preemption, 2017 102 Minnesota Law Review.* Available at: http://www.minnesotalawreview.org/2017/04/pot-printz-and-preemption/ (accessed April, 2017).

[B25] Gul W. Gul S. W. Chandra S. Lata H. Ibrahim E. A. ElSohly M. A. (2018). Detection and quantification of cannabinoids in extracts of cannabis sativa roots using LC-MS/MS. *Planta Med.* 84 267–271. 10.1055/s-0044-100798 29359294

[B26] Hampson A. Axelrod J. Grimaldi M. (2003). *Cannabinoids as Antioxidants and Neuroprotectants* U.S. Patent No. 6630507 Washington, DC: United States Patent Office.

[B27] Hanus L. Tchilibon S. Ponde D. E. Breuer A. Fride E. Mechoulam R. (2005). Enantiometeric cannabidiol derivatives: synthesis and binding to cannabinoid receptors. *Org. Biomol. Chem.* 3 1116–1123. 10.1039/b416943c 15750656

[B28] Hoffman D. Palumbo F. B. Yang Y. T. (2018). *Will the FDA’s Approval of Epidiolex Lead to Rescheduling Marijuana?* Available at: https://www.healthaffairs.org/do/10.1377/hblog20180709.904289/full/ (accessed September 24 2018).

[B29] Jefferson B. (2018). *Marijuana Enforcement,” in Memorandum for All US Attorneys.* Available at: https://www.justice.gov/opa/press-release/file/1022196/download (accessed September 24 2018).

[B30] Johnson R. (2018). *Hemp as an Agricultural Commodity.* Washington, DC: Congressional Research Service.

[B31] Johnston G. (2018). *Study to Examine Possible Effects of Cannabis Compound for Common Movement Disorder.* San Diego: UC San Diego Health.

[B32] Jones N. A. Glyn S. E. Akiyama S. Hill T. D. Hill A. J. Weston S. E. (2012). Cannabidiol exerts anti-convulsant effects in animal models of temporal lobe and partial seizures. *Seizure* 21 344–352. 10.1016/j.seizure.2012.03.001 22520455

[B33] Jones N. A. Hill A. J. Smith I. Bevan S. A. Williams C. M. Whalley B. J. (2010). Cannabidiol displays anti-epileptiform and anti-seizure properties in vitro and in vivo. *J. Pharmacol. Exp. Ther.* 332 569–577. 10.1124/jpet.109.159145 19906779 PMC2819831

[B34] Joy J. Watson S. Benson J. (eds) (1999). *Institute of Medicine, Marijuana and Medicine: Assessing the Science Base.* Washington, DC: National Academy Press.

[B35] Klieger S. B. Gutman A. Allen L. Pacula R. L. Ibrahim J. K. Burris S. (2017). Mapping medical marijuana: state laws regulating patients, product safety, supply chains and dispensaries. *Addiction* 112 2206–2216. 10.1111/add.13910 28696583 PMC5725759

[B36] Lee M. (2016). *Project CBD, Sourcing CBD: Marijuana Industrial Hemp & The vagaries of Federal Law.* Available at: https://www.projectcbd.org/about/cannabis-facts/sourcing-cbd-marijuana-industrial-hemp-vagaries-federal-law (accessed September 23 2018).

[B37] Maa E. Figi P. (2014). The case for medical marijuana in epilepsy. *Epilepsia* 55 783–786. 10.1111/epi.12610 24854149

[B38] Mead A. (2014). “International control of cannabis,” in *Handbook of cannabis*, ed. Pertwee R. (Oxford: Oxford University Press), 47.

[B39] Mechoulam R. Shani A. Edery H. Grunfeld Y. (1970). Chemical basis of hashish activity. *Science* 169 611–612. 10.1126/science.169.3945.611 4987683

[B40] Medical Board of California (2018). *Guidelines for the Recommendation of Cannabis for Medical Purposes.* Sacramento: Medical Board of California.

[B41] Milley J. (2018). *Which States do Not Require Medical Marijuana Testing and Why Not, 2018.* Available at: https://droseaz.com/which-states-do-not-require-medical-marijuana-testing-and-why-not/ (accessed October 14 2018).

[B42] Mister S. (2019). *Science, CBD, and the Prisoner’s Dilemma.* Rancho Palos Verdes, CA: Natural Products Insider.

[B43] Musto D. F. (1972). The Marihuana tax act of 1937. *Arch. Gen. Psychiatry.* 26 101–108. 10.1001/archpsyc.1972.01750200005002 4551255

[B44] National Conference on State Legislatures [NCSL] (2018). *National Conference on State Legislatures [NCSL].* Available at: http://www.ncsl.org/research/health/state-medical-marijuana-laws (accessed October 25 2018).

[B45] National Council of State Legislatures [NCSL] (2018). *National Council of State Legislatures [NCSL].* Available at: http://www.ncsl.org/research/about-state-legislatures/2018-state-legislative-session-calendar.aspx (accessed October 25 2018).

[B46] Ng R. (2015). *Drugs: From Discovery to Approval*, 3rd Edn Hoboken, NJ: John Wiley & Sons.

[B47] Pertwee R. G. (2004). “The pharmacology and therapeutic potential of cannabidiol,” in *Cannabinoids*, ed. Dicpe Marzo V. (New York, NY: Kluwer Academic/Plenum Publishers), 32–83.

[B48] Potter D. (2013). A review of the cultivation and processing of cannabis (Cannabis sativa L.) for production of prescription medicines in the UK. *Drug Test Anal.* 6 31–38. 10.1002/dta.1531 24115748

[B49] Potter D. (2014). “Cannabis horticulture,” in *Handbook of Cannabis*, ed. Pertwee R. (Oxford: Oxford University Press).

[B50] ProCon (2018a). *Edical Marijuana.* Available at: https://medicalmarijuana.procon.org/view.resource.php?resourceID=000881 (accessed October 13 2018).

[B51] ProCon (2018b). *Legal Recreational Marijuana States.* Available at: https://marijuana.procon.org/view.resource.php?resourceID=006868 (accessed October 13 2018).

[B52] Project CBD (2018). *Project CBD.* Available at: www.projectcbd.org/about/about-project-cbd (accessed September 23 2018).

[B53] Randall R. O’Leary A. (1998). *Marijuana Rx: The Patients’ Fight for Medicinal Pot.* New York, NY: Hachette Book Group.

[B54] Russo E. B. (2004). “History of cannabis as a medicine,” in *The Medicinal Uses of Cannabis and Cannabinoids*, eds Robson G. Whittle (London: Pharmaceutical Press).

[B55] Schlosser E. (1994). *Reefer Madness The Atlantic*. Available at: https://www.theatlantic.com/magazine/archive/1994/08/reefer-madness/303476/ (accessed October 25 2018).

[B56] Single Convention on Narcotic Drugs (1961). *Single Convention on Narcotic Drugs.* Available at: https://www.incb.org/documents/Narcotic-Drugs/1961-Convention/convention_1961_en.pdf (accessed October 25 2018).

[B57] The Medicalization of Cannabis (2009). *Wellcome Witnesses to Twentieth Century Medicine*, Vol. 40 London: Wellcome Trust Centre for the History of Medicine.

[B58] Todd G. (2012). *Medical Marijuana: The Supremacy Clause, Federalism, and the Interplay between State and Federal Laws.* Washington, DC: Congressional Research Service.

[B59] Uniform Controlled Substances Act (1994). *Uniform Controlled Substances Act.* Available at: http://www.uniformlaws.org/shared/docs/controlled%20substances/UCSA_final%20_94%20with%2095amends.pdf (accessed September 24 2018).

[B60] University of Mississippi (2018). *Marijuana Research.* Available at: https://pharmacy.olemiss.edu/marijuana/ (accessed September 24 2018).

[B61] US Attorney Marshall Letter to Rep. Blumenauer (2018). *US Attorney Marshall Letter to Rep. Blumenauer.* Available at: http://votehemp.com/PDF/Ltr_to_Earl_Blumenauer_110713.pdf (accessed September 24 2018).

[B62] US Dep’t of Health and Human Services and US FDA (2016). *Botanical Drug Development: Guidance for Industry.* Silver Spring: US FDA.

[B63] Vantreese V. L. (2002). Hemp support: evolution in EU regulation. *J. Industr. Hemp* 7 17–31. 10.1300/j237v07n02_03

[B64] Vogelstein F. Boy Interrupted Wired Magazine (2015). Available at: https://www.wired.com/2015/07/medical-marijuana-epilepsy/ (accessed September 23 2018).

[B65] Wang M. Wang Y. H. Avula B. Radwan M. M. Wanas A. S. van Antwerp J. (2016). Decarboxylation study of acidic cannabinoids: a novel approach using ultra-high-performance supercritical fluid chromatography/photodiode array-mass spectrometry. *Cannabis Cannabinoid Res.* 1 262–271. 10.1089/can.2016.0020 28861498 PMC5549281

[B66] Yeh B. T. (2012). *The Controlled Substances Act: Regulatory Requirements.* Washington, DC: Congressional Research Service.

